# Case Report: Staged limb salvage and delayed ankle arthrodesis for chronic distal tibial osteomyelitis after failed fixation

**DOI:** 10.3389/fsurg.2026.1874274

**Published:** 2026-08-06

**Authors:** Abdikarim Mahad Ahmed, Abdulkhalek Hassan Mohamed, Osman Mohamed Abdulkarim, Mohamed Abdullahi Omar, Abdullahi Khalif Ali, Nur Adam Mohamed, Halwo Bashir Adan Kainan, Mohamed Mukhtar Mohamed

**Affiliations:** 1 Department of Orthopaedics and Traumatology, Mogadişu Somali Türkiye Recep Tayyip Erdoğan Eğitim ve Araştırma Hastanesi, Mogadishu, Somalia; 2 Department of General Surgery, Mogadişu Somali Türkiye Recep Tayyip Erdoğan Eğitim ve Araştırma Hastanesi, Mogadishu, Somalia; 3 Department of Psychiatry, Mogadişu Somali Türkiye Recep Tayyip Erdoğan Eğitim ve Araştırma Hastanesi, Mogadishu, Somalia; 4 Department of Cardiovascular Surgery, Mogadişu Somali Türkiye Recep Tayyip Erdoğan Eğitim ve Araştırma Hastanesi, Mogadishu, Somalia; 5 Faculty of Medicine and Surgery, Salaam University, Mogadishu, Somalia

**Keywords:** distal tibia, chronic osteomyelitis, limb salvage, ankle arthrodesis, external fixation, antibiotic-impregnated cement spacer, MRSA, case report

## Abstract

Chronic osteomyelitis of the distal tibia following failed fracture fixation is a challenging condition because of limited soft-tissue coverage, compromised vascularity, persistent infection, and difficulty achieving stable reconstruction. We report the case of a 34-year-old female who developed chronic post-traumatic osteomyelitis of the distal tibia after failed initial management of a distal tibial fracture. She presented with persistent pain, swelling, inability to bear weight, and a discharging sinus. Radiographic evaluation showed persistent distal tibial fracture pathology and failure of the initial fracture stabilization strategy, while the diagnosis of chronic osteomyelitis was supported by clinical findings, elevated inflammatory markers, intraoperative findings, and deep intraoperative cultures. Microbiological culture identified methicillin-resistant *Staphylococcus aureus* from deep intraoperative bone and soft-tissue specimens obtained during surgical debridement; no superficial wound swabs were used, and the patient was not receiving suppressive antibiotic therapy before sampling. A staged limb salvage approach was performed, beginning with radical debridement, removal of infected hardware, temporary external fixation, antibiotic-impregnated cement spacer placement, and targeted antibiotic therapy. After resolution of sinus drainage, complete wound healing, improvement of inflammatory markers, and control of infection, definitive reconstruction was performed with delayed ankle arthrodesis using an intramedullary nail. At 6 months after definitive ankle arthrodesis, corresponding to approximately 14 months after the original injury, the patient remained free of clinically evident recurrent infection, was ambulating independently without assistive devices, had a Visual Analogue Scale pain score of 0–1, and showed radiographic progression toward ankle fusion without implant failure. This case highlights the importance of staged infection control before definitive reconstruction and demonstrates that delayed ankle arthrodesis can provide a stable and functional limb in complex post-traumatic distal tibial osteomyelitis when joint preservation is not feasible. Management was undertaken in a resource-limited setting, where restricted access to advanced imaging and specialized reconstructive options influenced clinical decision-making and necessitated a pragmatic staged limb salvage approach.

## Introduction

Distal tibial and tibial plafond fractures remain difficult injuries to manage in orthopaedic trauma. Because of the vulnerable soft-tissue envelope around the distal tibia, these fractures are associated with wound complications, infection, delayed union, non-union, and poor functional outcomes after operative fixation (1, 2). When fixation fails or soft-tissue problems are not adequately controlled, the risk of deep infection and long-term disability increases.

Chronic post-traumatic osteomyelitis is one of the most serious complications after failed fracture fixation. It is often associated with persistent infection, devitalized bone, sequestrum formation, chronic inflammation, and biofilm-forming organisms. These factors make treatment difficult, as systemic antibiotics alone are often insufficient to eradicate infection (3, 4). Therefore, successful management usually requires both surgical and antimicrobial treatment.

Treatment of chronic osteomyelitis of the distal tibia is especially challenging and is commonly performed in stages. The first stage is directed toward infection control and usually includes radical debridement, removal of infected or failed hardware, deep tissue or bone sampling for microbiological diagnosis, dead-space management, and restoration of stability when needed (5, 6). In infected distal tibial fractures, temporary external fixation may be useful to maintain alignment while infection control and soft-tissue recovery are achieved (7).

Local antibiotic delivery is an important adjunct in orthopaedic infection management, as it can provide high antibiotic concentrations at the infected site while also helping to manage dead space (8, 9). Definitive reconstruction is generally delayed until there is clinical and laboratory evidence that infection has been controlled. When preservation of the ankle joint is no longer feasible because of severe joint destruction, instability, bone loss, or infection-related damage, ankle arthrodesis remains a practical limb salvage option. Although it sacrifices ankle motion, it can provide pain relief, stability, and functional ambulation (7, 10).

## Case description

**Figure 1:**
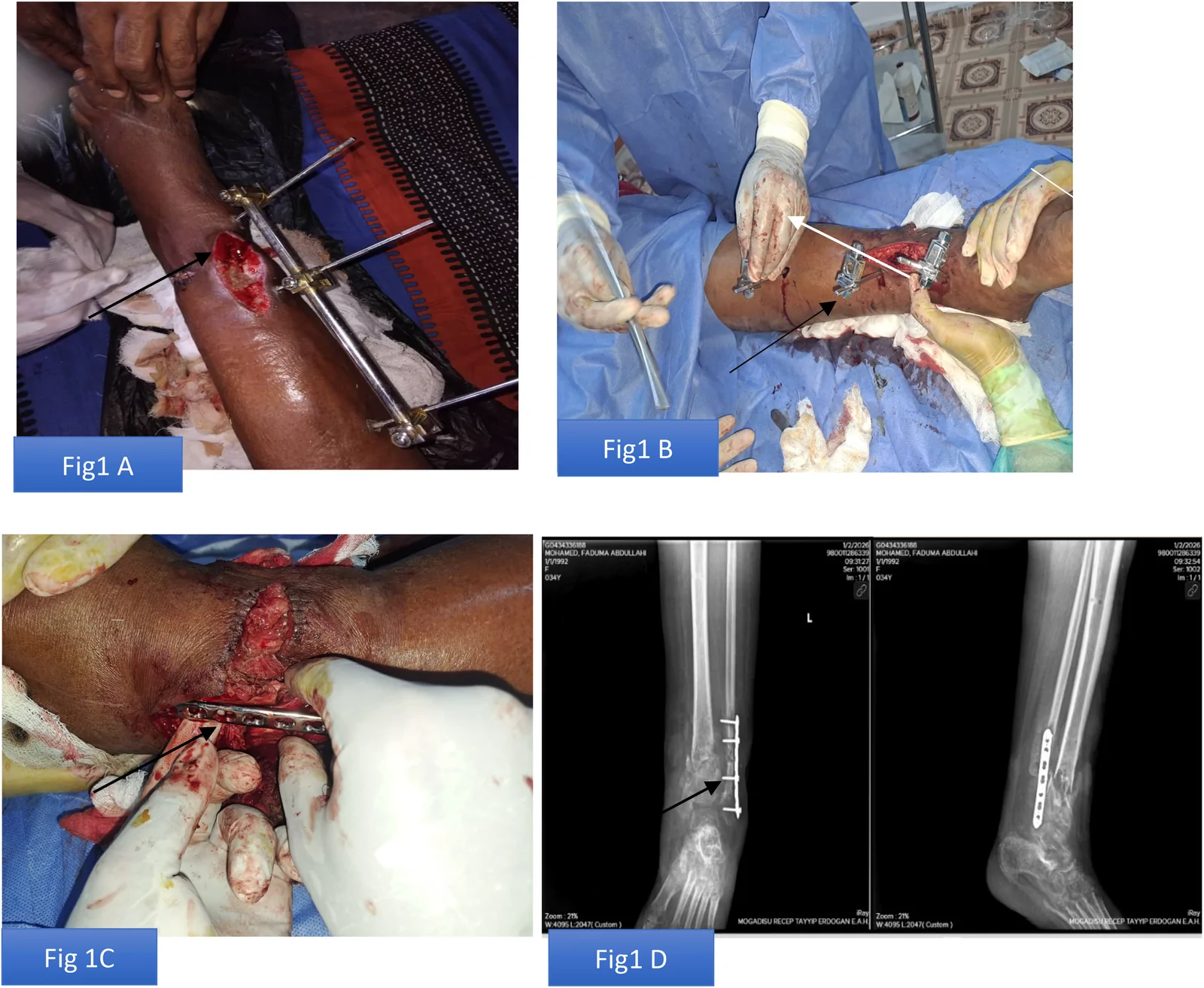
Initial management and early complications following distal tibial fracture fixation of the left leg. **(A)** Clinical appearance of the left leg after initial external fixation at the referring hospital. The arrow indicates the surgical incision/wound related to treatment and should not be interpreted as an open fracture wound at presentation. The original injury was a closed distal tibial fracture. **(B)** Clinical appearance during removal of the external fixator at the referring hospital. The arrow indicates the previous external-fixator/pin-site region. **(C)** Clinical photograph after conversion to internal fixation, demonstrating wound breakdown, exposed fibular fixation, and extensive wound contamination associated with fixation failure. The arrow indicates the affected wound area. **(D)** Radiograph obtained after conversion to internal fixation, demonstrating fibular plate/screw fixation without direct stabilization of the distal tibial fracture. The arrows indicate the fibular fixation.

A 34-year-old female presented to our outpatient department with persistent pain, swelling, and inability to bear weight on the left lower limb following a road traffic accident that resulted in a closed distal left tibial fracture. The fracture had initially been managed at a referring centre with external fixation, followed by conversion to open reduction and internal fixation with plate and screws. There was no history or documentation indicating that the fracture was open at the time of injury. The wound seen in Figure 1A represents a surgical incision/wound related to the initial management at the referring hospital and should not be interpreted as an open fracture wound at presentation.

Clinical and radiographic findings from the patient's initial management at the referring hospital are shown in Figure 1. The clinical sequence at the referring hospital included initial external fixation, subsequent removal of the external fixator, and conversion to internal fixation. Figure 1A shows the initial external fixation stage and the surgical incision/wound related to treatment, which should not be interpreted as an open fracture wound at presentation. Figure 1B shows the clinical appearance during removal of the external fixator. Figure 1C demonstrates the clinical wound complication after conversion to internal fixation, including wound breakdown and exposed fibular fixation. Figure 1D shows the corresponding radiograph after internal fixation, demonstrating fibular fixation without adequate stabilization of the distal tibial fracture. The complete clinical timeline from the initial injury through definitive reconstruction and follow-up is summarized in Table 1.

**Table 1:** Timeline.

Time from injury	Clinical event	Related figure
Day 0	Road traffic accident resulting in a closed distal left tibial fracture	—
Day 1	Temporary external fixation was applied at the referring hospital	Figure 1A
Week 5	The external fixator was removed, and the patient underwent conversion to internal fixation. A fibular plate and screws were applied without adequate direct stabilization of the distal tibial fracture	Figures 1B,D
Month 3	Wound breakdown, exposure of the fibular fixation, and persistent wound discharge developed	Figure 1C
Month 4	Progressive local infection developed, with formation of a discharging sinus	Figure 2A
Month 5	Fixation failure progressed, with worsening infection and development of chronic post-traumatic osteomyelitis	Figure 2B
Month 6	The patient presented to our tertiary center with pain, swelling, a discharging sinus, infected hardware, and inability to bear weight	Figures 2A,B
Month 6	First-stage surgery was performed, including radical debridement, removal of infected hardware, deep bone and soft-tissue sampling, placement of an antibiotic-loaded PMMA spacer, and temporary delta-frame external fixation	Figures 3A,B
Months 6–8	The patient received 6 weeks of culture-directed intravenous antibiotic therapy followed by 2 weeks of oral step-down therapy, with progressive wound healing and soft-tissue recovery	—
Month 8	Definitive ankle arthrodesis was performed using an intramedullary nail after clinical and laboratory evidence of infection control	—
Month 11	At 3 months after arthrodesis, the patient demonstrated reduced pain and improved functional status	—
Month 14	At 6 months after arthrodesis, the patient demonstrated complete wound healing, no recurrent sinus or drainage, independent ambulation, maintained alignment, and radiographic progression toward fusion	Figure 4A

## Diagnostic assessment

At presentation to our tertiary center, the working diagnosis was chronic post-traumatic osteomyelitis of the distal tibia associated with failed fracture stabilization. The patient had previously undergone external fixation at the referring hospital, followed by removal of the external fixator and conversion to internal fixation with plate and screws. At the time of presentation to our institution, the external fixator was no longer present. The patient presented with failed internal fixation, wound breakdown, persistent drainage, exposed/infected hardware, and inability to bear weight. The fixation did not allow functional weight-bearing because of pain, infection, soft-tissue compromise, exposed/infected hardware, and inadequate stabilization of the distal tibial fracture.

**Figure 2:**
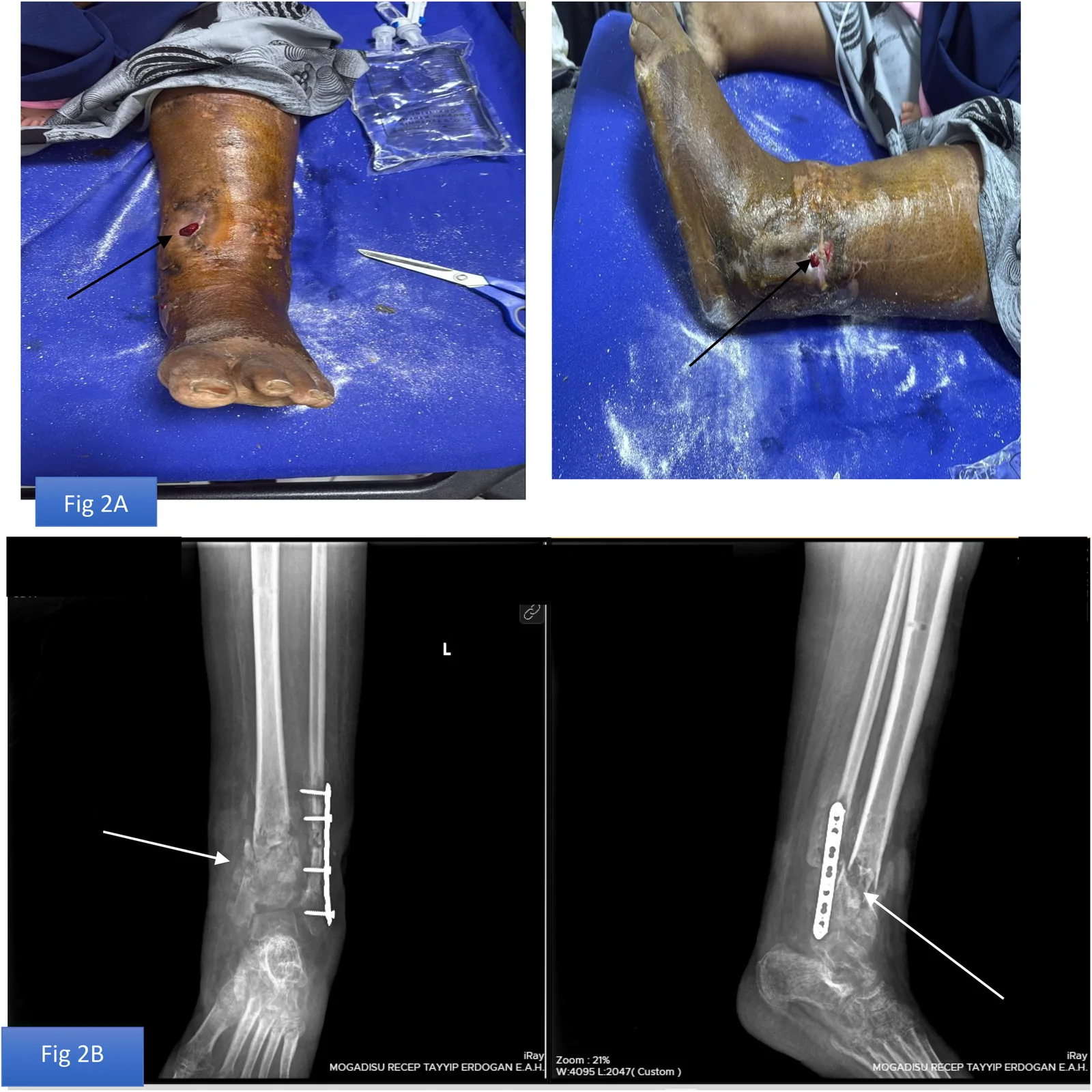
Clinical and radiographic findings at presentation to our institution. **(A)** Clinical image of the left leg showing a discharging sinus over the distal tibia with purulent discharge, suggestive of infection. The arrow indicates the discharging sinus tract. **(B)** Radiograph obtained at presentation to our tertiary center showing the previously performed fibular fixation and persistent distal tibial fracture pathology. The arrow indicates the distal tibial fracture region.

Radiographs obtained at presentation to our tertiary center showed the previously performed fibular fixation and persistent distal tibial fracture pathology (Figure 2B). Although plain radiographs alone did not clearly demonstrate interval progression compared with earlier imaging, the diagnosis of chronic post-traumatic osteomyelitis was supported by wound breakdown, persistent drainage, exposed/infected hardware, a discharging sinus, elevated inflammatory markers, intraoperative findings of infected and necrotic tissue, and deep intraoperative cultures positive for MRSA. Laboratory investigations revealed elevated inflammatory markers, including an erythrocyte sedimentation rate of 35 mm/hr and a C-reactive protein level of 30 mg/L.

No superficial wound swabs were used, and the patient had not received suppressive antibiotics before sampling. Microbiological culture identified methicillin-resistant *Staphylococcus aureus* (MRSA), which guided subsequent targeted intravenous antibiotic therapy. Based on the clinical presentation, radiographic findings, inflammatory markers, and microbiological culture results, the final diagnosis was chronic post-traumatic osteomyelitis of the distal tibia associated with failed fracture stabilization.

## Therapeutic intervention

**Figure 3:**
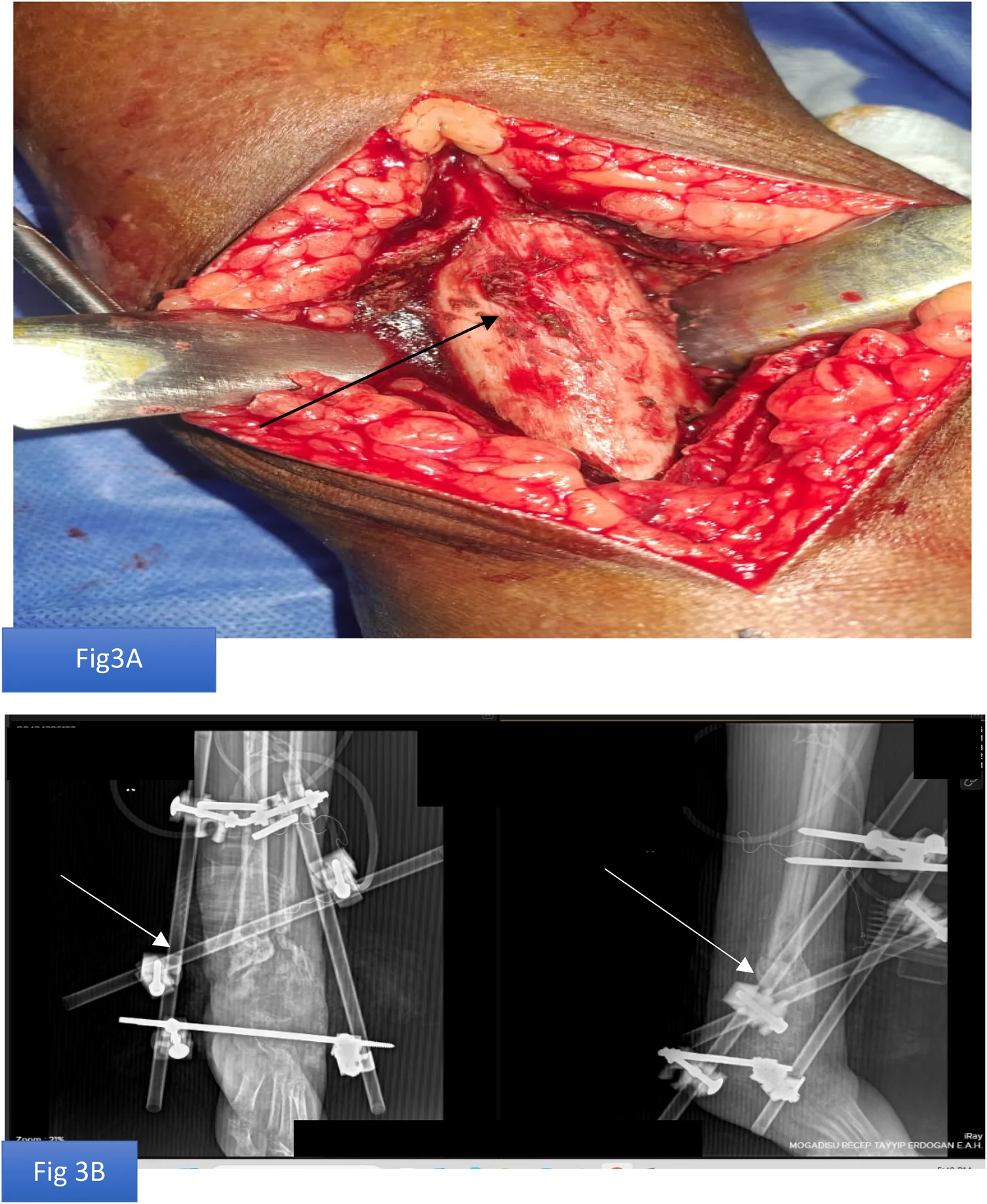
First-stage surgical debridement and temporary stabilization of the left leg. **(A)** Intraoperative image of the left leg demonstrating radical debridement. The arrows indicate necrotic bone and infected soft tissue removed during debridement. **(B)** Postoperative image of the left leg showing delta-frame external fixation. The arrows indicate the external fixator components used for temporary limb stabilization.

A staged limb salvage strategy was implemented. The first stage consisted of radical surgical debridement with complete excision of infected and necrotic tissues, together with removal of all previously implanted hardware. Intraoperative findings are shown in Figure 3A, where the arrows indicate necrotic bone and infected soft tissue removed during debridement. Temporary stabilization of the limb was achieved using a delta-frame external fixation construct (Figure 3B), where the arrows indicate the external fixator components used for temporary limb stabilization.

Following thorough debridement, an antibiotic-loaded polymethylmethacrylate (PMMA) cement spacer was prepared intraoperatively under sterile conditions using a hand-mixed technique. For each 40 g batch of PMMA bone cement, 2 g of vancomycin and 0.5 g of gentamicin were incorporated to provide high local antibiotic concentrations with broad-spectrum antimicrobial coverage, including activity against methicillin-resistant *Staphylococcus aureus* (MRSA). The antibiotics were uniformly mixed with the cement powder prior to polymerization to support local antibiotic release.

The spacer was then inserted into the defect to facilitate local infection control, dead-space management, and temporary structural support. Following radical debridement, a clinically significant osseous defect remained in the distal tibial and ankle region. However, the exact longitudinal measurement of the defect was not prospectively documented, and a reliable retrospective measurement could not be obtained from the available non-calibrated radiographs and intraoperative photographs. No commercially premixed antibiotic-loaded cement was used.

The patient received culture-directed intravenous antibiotic therapy targeting MRSA for 6 weeks following surgical debridement and receipt of intraoperative culture results. This was followed by a 2-week course of oral antibiotic therapy as step-down treatment to consolidate infection control. Definitive reconstruction was undertaken approximately 8 weeks after the first-stage debridement, once sinus drainage had resolved, the wound had healed, inflammatory markers had improved, and there was no clinical evidence of ongoing infection.

## Follow-up and outcomes

At the final assessment, performed 6 months after definitive ankle arthrodesis and approximately 14 months after the original injury, the patient demonstrated satisfactory short-term clinical recovery, defined by complete wound healing, absence of recurrent sinus or wound drainage, absence of local tenderness, no clinical evidence of recurrent infection, improved pain control with a VAS pain score of 0–1, and independent household and community ambulation without assistive devices.

**Figure 4:**
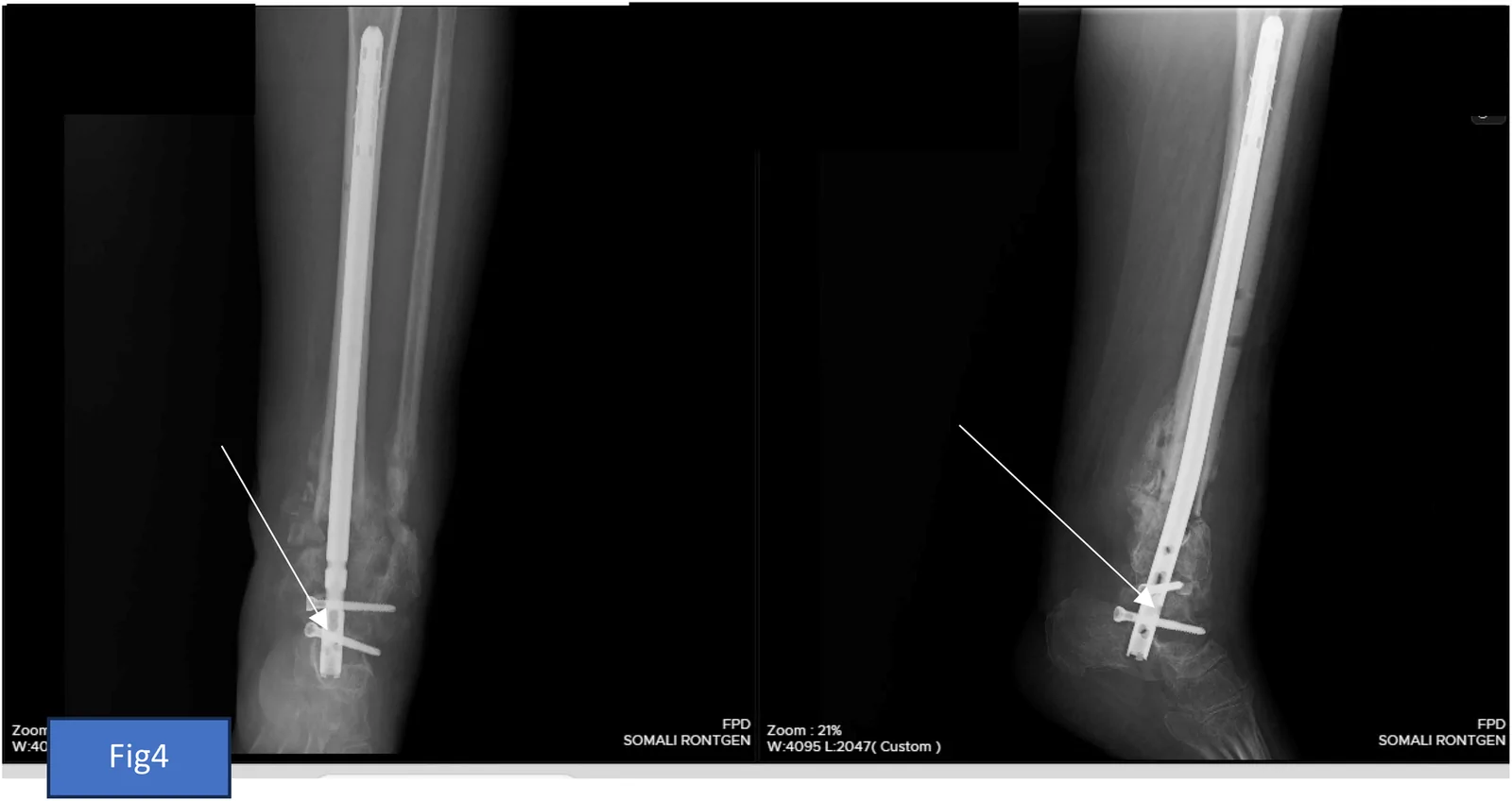
Definitive reconstruction with ankle arthrodesis. **(A)** Anteroposterior and lateral radiographs obtained 6 months after definitive ankle arthrodesis demonstrating intramedullary nail fixation with maintained alignment and progression toward fusion. The arrow indicates the ankle arthrodesis site.

The patient had a stable, plantigrade limb with restoration of functional ambulation. Radiographs obtained 6 months after definitive ankle arthrodesis demonstrated maintained alignment and progression toward fusion, with no evidence of implant loosening or recurrent osteolytic changes (Figure 4A).

Clinical and plain-radiographic assessment demonstrated satisfactory progression toward fusion. However, computed tomography was not available because of resource limitations. CT is important after ankle arthrodesis because it can more accurately assess osseous bridging across the fusion site, particularly when implant overlap and complex anatomy limit the interpretation of plain radiographs. Therefore, complete fusion could not be definitively confirmed in this case, and the radiographic findings should be interpreted as progression toward fusion rather than CT-confirmed union.

Pain and functional outcomes were assessed using the Visual Analog Scale (VAS) and the American Orthopaedic Foot and Ankle Society (AOFAS) Ankle-Hindfoot Score. The VAS is a patient-reported measure of pain severity ranging from 0, indicating no pain, to 10, indicating the worst imaginable pain. The AOFAS Ankle-Hindfoot Score is a composite clinical assessment incorporating pain, function, and alignment and is therefore not considered a purely patient-reported outcome measure. Additional validated patient-reported outcome measures, including the Foot and Ankle Ability Measure (FAAM), Foot Function Index (FFI), and EuroQol-5 Dimension (EQ-5D), were not prospectively collected and could not be reliably reconstructed retrospectively.

Follow-up assessments demonstrated progressive resolution of infection, including cessation of sinus discharge, resolution of local tenderness, reduction in pain and swelling, complete wound healing, and restoration of functional ambulation without assistive devices. No recurrent wound breakdown or delayed wound healing was observed after definitive ankle arthrodesis. Serial radiographs demonstrated maintained alignment and continued progression toward fusion following staged debridement, antibiotic-loaded cement spacer placement, and definitive ankle arthrodesis.

The 6-month post-arthrodesis assessment represented the longest available follow-up at the time of manuscript preparation. This duration is insufficient to confirm long-term eradication of infection, mature osseous fusion, implant survival, or sustained functional recovery. Therefore, the findings should be interpreted as short-term outcomes, and continued clinical and radiographic surveillance is required.

Progressive improvement in pain intensity and functional status was observed following staged infection control and definitive ankle arthrodesis. The short-term outcome demonstrated restoration of limb stability, pain reduction, and functional ambulation in the context of chronic post-traumatic distal tibial osteomyelitis.

## Patient perspective

The patient reported improvement in pain, limb stability, and ability to ambulate after staged treatment. She expressed satisfaction with limb preservation and functional recovery.

## Discussion

Distal tibial fractures remain difficult injuries to manage, particularly in high-energy trauma and cases requiring operative fixation. The distal tibia has a relatively thin soft-tissue envelope and limited vascularity, which can increase the risk of wound breakdown, deep infection, fixation failure, delayed union, and non-union (1, 2). In the present case, progressive infection was supported by wound breakdown, persistent drainage, exposed hardware, sinus formation, elevated inflammatory markers, intraoperative findings of infected and necrotic tissue, and deep intraoperative cultures positive for methicillin-resistant *Staphylococcus aureus*.

Chronic osteomyelitis is challenging because infection may persist within necrotic bone, sequestra, and biofilm-containing tissue (3, 4). These factors reduce the effectiveness of systemic antibiotics alone and make surgical infection control essential. In this patient, radical debridement, removal of infected hardware, and creation of a healthier biological environment were necessary before definitive reconstruction could be considered (5, 6).

A staged approach was selected because it allowed infection control and reconstruction to be addressed separately. The first stage focused on reducing the bacterial burden, removing infected and necrotic tissue, removing failed implants, stabilizing the limb, and allowing soft-tissue recovery (5, 6). Temporary external fixation was used to maintain alignment while avoiding additional internal hardware placement in an infected field (7).

Local antibiotic delivery was also an important part of treatment. The antibiotic-loaded PMMA cement spacer provided high local antibiotic concentrations, helped manage dead space, and offered temporary structural support after debridement (8, 9). This was combined with culture-directed systemic antibiotic therapy based on the MRSA isolate.

The timing of definitive reconstruction was guided by clinical and laboratory evidence of infection control. Definitive reconstruction was delayed until sinus drainage had resolved, the wound had healed, inflammatory markers had improved, and there was no clinical evidence of ongoing infection (5, 6). A long intramedullary nail was chosen for ankle arthrodesis because it provided stable load-sharing fixation across the fusion site while bypassing the compromised distal tibial segment affected by chronic infection, bone destruction, and previous fixation failure. At 6-month follow-up, radiographs showed maintained alignment and progression toward fusion, although complete fusion could not be confirmed with CT because of resource limitations.

Joint preservation was not considered feasible in this case. Chronic infection and fixation failure had caused extensive destruction of the distal tibial articular region, compromised bone stock, soft-tissue compromise, and loss of a stable, reconstructable ankle joint. In this setting, procedures aimed at preserving ankle motion were unlikely to provide durable stability, reliable infection control, or a satisfactory functional outcome. Therefore, delayed ankle arthrodesis was selected as the most appropriate limb salvage option to achieve pain relief, mechanical stability, and functional weight-bearing.

Recent literature supports the use of staged and individualized limb-salvage strategies for complex infections involving the distal tibia and ankle. Mascio et al. reported a retrospective series of 17 patients with distal tibial and ankle osteoarticular infections managed using different combinations of radical debridement, antibiotic-loaded cement spacers, external fixation, arthrodesis, bone reconstruction, and soft-tissue procedures. Their findings emphasize that successful limb salvage requires adequate infection control, stable reconstruction, and prolonged follow-up because complications and additional procedures remain common (11). Comisi et al. also described tibiotalocalcaneal arthrodesis using a retrograde intramedullary nail for severe talar destruction caused by tuberculosis. Although the infectious etiology differed from the post-traumatic MRSA osteomyelitis in our patient, their case supports hindfoot arthrodesis as a salvage option when extensive infectious destruction makes preservation of the ankle joint impractical (12). These reports are consistent with our staged approach, in which radical debridement, temporary stabilization, local antibiotic delivery, and delayed definitive arthrodesis were used to achieve infection control and restore a stable, plantigrade limb.

Amputation was considered but was not favored. The patient was young, the limb remained neurovascularly intact, infection control appeared achievable after radical debridement, and preservation of a functional plantigrade limb was possible. In addition, access to advanced prosthetic fitting, rehabilitation services, and long-term prosthesis maintenance may be limited in resource-constrained settings. For these reasons, limb salvage was considered more appropriate than early amputation, provided that infection control and mechanical stability could be achieved.

The resource-limited setting significantly influenced both diagnosis and management. Preoperative CT could have provided a more detailed assessment of cortical destruction, sequestra, articular involvement, and the extent of bone loss, thereby supporting surgical debridement and reconstruction planning. Advanced imaging, such as MRI to define the full extent of osteomyelitis and CT to confirm complete fusion, was not readily available.

Access to microsurgical soft-tissue reconstruction, vascularized bone grafting, circular-frame bone transport, advanced prosthetic rehabilitation, and long-term prosthesis maintenance may also be restricted. As a result, management relied on careful clinical assessment, plain radiography, inflammatory markers, deep intraoperative cultures, radical debridement, local antibiotic delivery, temporary external fixation, and delayed definitive stabilization. This case therefore highlights how established principles of infection control and staged reconstruction can be adapted to a resource-limited setting.

The value of this report does not lie in introducing a novel surgical protocol, because staged debridement, temporary stabilization, local antibiotic delivery, and delayed reconstruction are established principles in the management of chronic osteomyelitis. Rather, this case demonstrates the practical clinical workflow and adaptation of this established strategy in a resource-limited setting. Because this report describes a single patient, no conclusions regarding the general efficacy or safety of this treatment strategy can be drawn.

In this patient, the structured staged limb-salvage approach was associated with a favorable short-term outcome. At the 6-month follow-up, the patient had no clinical evidence of recurrent infection, showed complete wound healing, had improved pain control, and was able to ambulate independently without assistive devices. However, this follow-up duration remains short for chronic osteomyelitis, particularly after the reintroduction of internal metalwork. Longer follow-up is required to assess the durability of fusion, late infection recurrence, implant-related complications, and sustained functional recovery.

This report has several important limitations. First, it describes a single patient, which limits the generalizability of the findings. Second, follow-up was limited to 6 months after definitive ankle arthrodesis, corresponding to approximately 14 months after the original injury. This duration is insufficient to confirm mature osseous fusion, durable eradication of infection, long-term implant survival, or sustained functional recovery.

Third, CT imaging was not available because of resource limitations. CT would have provided more accurate characterization of the initial cortical and articular destruction, sequestra, residual osseous defect, and postoperative osseous bridging. Consequently, complete fusion could not be definitively confirmed, and the radiographic findings should be interpreted as progression toward fusion rather than CT-confirmed union.

Fourth, the exact longitudinal measurement of the osseous defect was not prospectively documented and could not be reliably reconstructed from the available non-calibrated images. Fifth, although VAS and AOFAS assessments were available, additional validated patient-reported outcome measures, including FAAM, FFI, and EQ-5D, were not prospectively collected. Finally, late recurrent infection and implant-related complications remain possible, particularly after reintroduction of internal metalwork into a previously infected anatomical region. Longer clinical and radiological surveillance is therefore required.

## Conclusion

This case shows that chronic post-traumatic distal tibial osteomyelitis after failed fixation can be successfully managed with a careful staged limb salvage approach. In this patient, radical debridement, removal of infected hardware, local antibiotic delivery, temporary stabilization, and delayed reconstruction allowed infection control before definitive fixation. Because the ankle joint was no longer suitable for preservation, delayed ankle arthrodesis provided a stable, pain-controlled, and functional limb. The case also highlights that, even in resource-limited settings, good outcomes may be achieved when the basic principles of infection control, soft-tissue recovery, and mechanical stability are followed.

## Data Availability

The original contributions presented in this case report are included in the article. Further inquiries can be directed to the corresponding author.
